# Supplementary material for: scGraphformer: unveiling cellular heterogeneity and interactions in scRNA-seq data using a scalable graph transformer network
Source: Commun Biol. 2024 Nov 8;7:1463. doi: 10.1038/s42003-024-07154-w (PMC11543810; doi:10.1038/s42003-024-07154-w)
Supplement: Supplementary file 2 — Supplementary Information [file 42003_2024_7154_MOESM2_ESM.pdf]

Supplementary Information for scGraphformer: Unveiling Cellular Heterogeneity and Interactions in scRNA-seq Data using a Scalable Graph Transformer Network

Xingyu Fan[1], Jiacheng Liu[1*], Yaodong Yang[1], Chunbin Gu[1], Yuqiang Han[1], Bian Wu[2], Yirong Jiang[3], Guangyong Chen[2*], Pheng-Ann Heng[1]

[1]Department of Computer Science and Engineering, The Chinese University of Hong Kong, Hong Kong, China.
[2]Zhejiang Lab, Hangzhou, China.
[3]Department of Chemistry, Zhejiang University, Hangzhou, China.

*Corresponding author(s). E-mail(s): jiachengliu@cuhk.edu.hk; gychen@zhejianglab.com;
Contributing authors: xyfan@link.cuhk.edu.hk; yangyaodong@link.cuhk.edu.hk; cbgu@cuhk.edu.hk; yuqianghan@cuhk.edu.hk; wub@zhijianglab.com; yrjiang@zju.edu.cn; pheng@cse.cuhk.edu.hk;

Appendix A Cell network learning

Shown in Fig A1, the scGraphformer further incorporates all-pair cell network learning structure to update the topological structure of the cell relationship network dynamically. This module consists of an aggregation module and a novel computation mechanism that combines the learned gene-gene interactions from the multi-head attention part. And the dynamic updating of the cell relationship network is a critical aspect of scGraphformer. The aggregation module enhances the modeling of higher-order relationships, contributing to an improved comprehension of cellular connectivity patterns. By incorporating learned attention scores (i.e. Query and Key) and aggregated features, this process involves continuously assigning and adjusting edge

connections between cells. Consequently, the constructed network accurately reflects the underlying biological proximity and inter-cellular interactions.

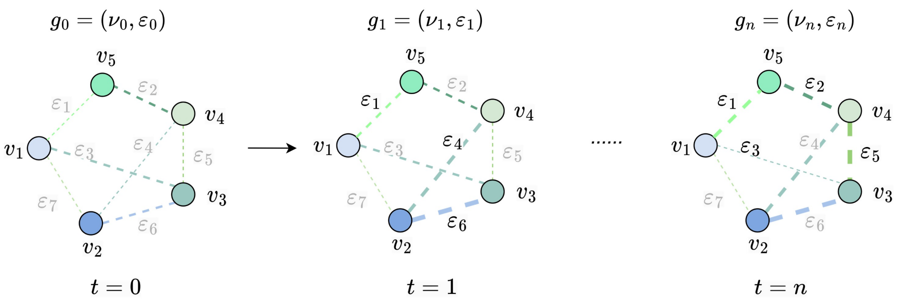

Fig. A1 All-pair cell network learning scheme. The scGraphformer learns all-pair cell network at each learning step. Here we simulate the learning process at each layer. In layer t=0, the scGraphformer establish the network connecting all cells which is g_0. In layer t=1 (if we set the learning layer more than 0), the scGraphformer updates the network based on g_0 and obtains g_1. The process will iteratively update in the following layers. The thickness of the lines indicates the degree of relationship between cells.

Appendix B Dataset details

We have evaluated scGraphformer on 31 datasets which are divided into intra-datasets, inter-datasets, and large-scale atlas. And we mainly collected the intra-datasets and inter-datasets from Zenodo repository [1]. The detailed information of each scRNA-seq dataset including their cell number, gene number, cell type number and protocol is in showed in Table B1.

Appendix C Results on intra-datasets

All of comparison results are shown in Table C2. Each method is running five times and the value represents its mean accuracy and standard deviation. In Table C5, it shows all five experiments' results on scGraphformer. Table C6 shows comparison of each method on baron human datasets, and each column represents cell type. Besides accuracy, we have also compared the F1-score and Cohen's Kappa score in intra-datasets evaluation which are shown in Table C3 and Table C4.

Table B1 Overview of the datasets for evaluation. We collected 20 datasets for intra-experiments and collected datasets from seven protocols for iter experiments. And we also collected 4 atlas for evaluating our performance on large-scale experiments.

Dataset	# Cell	# Gene	# Cell Type	Protocol	Reference
		Intra Dataset			
Adam	3660	23797	8	Drop-seq	[2]
Bach	23184	19965	8	10X	[3]
Klein	2717	24047	4	inDrop	[4]
lake	3042	25051	16	Fluidigm C1	[5]
Tosches turtle	18664	23500	15	Drop-seq	[6]
Segerstolpe	2133	22757	13	Smart-seq2	[7]
Young	5685	33658	11	10X	[8]
Zheng 68K	65943	20387	11	10X	[9]
Deng	268	22431	6	Smart-seq2	[10]
Darmanis	466	22088	9	SMARTer	[11]
usoskin	622	25334	4	STRT-Seq	[11]
campLiver	777	19020	7	SMARTer	[12]
Baron Mouse	1886	14861	13	inDrop	[13]
Muraro	2122	18915	9	CEL-Seq2	[14]
AMB	12832	42625	110	SMART-Seq v4	[15]
Baron Human	8569	17499	14	inDrop	[13]
Xin	1449	33889	4	SMARTer	[16]
TM (Tabula Muris)	54865	19791	55	10X Genomics	[17]
campbell	21086	26774	21	Drop-Seq	[18]
zilionis	34558	41861	9	inDrop	[19]
		Inter Dataset			
PBMC 10Xv2	23154	22280	9	10X	[20]
PBMC Drop-Seq	23154	19922	9	Drop-Seq	[20]
PBMC inDrop	21832	17159	7	inDrop	[20]
PBMC CEL-Seq	19754	20041	7	CEL-Seq	[20]
PBMC 10Xv3	19690	21905	8	10Xv3	[20]
PBMC Seq-Well	18966	21059	7	Seq-Well	[20]
PBMC Smart-Seq2	18886	22617	6	Smart-Seq2	[20]
		Large-scale Dataset			
Human Neocortex Atlas	638941	30265	19	SMART-seq, 10Xv3	[21]
Covid-19 Atlas	1462702	27714	64	10X	[22]
Zeisel	145954	27998	10	10X	[23]
Rosenberg	133435	26894	10	SPLiT-seq	[24]

Appendix D Results on inter-datasets

We evaluate our methods on inter-datasets from seven protocols, which are also called cross-platform experiments. Table D7 shows the detailed results of each experiment on accuracy score while performing inter-experiments using accuracy metric. Each experiment is also executed 5 times. Besides the situation that the reference is as same as the query datasets, there are 42 experiments for each method. And we compute their

Table C2 Comparison of methods on intra-experiments using Accuracy. The values represent mean accuracy and standard deviation over five independent repeating experiments. And scGraphformer outperforms other methods on most of datasets which has been shown in bold text.

method	scGraph -former	scVI	Cell -Typist	scmap -cell	scmap -cluster	ACTINN	scBal -ance	scBert (ZS)	scBert (FT)	TOSICA	scType
Baron Human	98.95 ± 0.25	96.25 ± 0.58	97.29 ± 0.35	97.81 ± 0.17	97.37 ± 0.27	93.75 ± 1.84	98.38 ± 0.19	26.08 ± 0.00	91.19 ± 0.00	97.00 ± 0.00	94.17 ± 0.00
campLiver	99.23 ± 0.84	92.56 ± 3.73	98.08 ± 1.15	93.46 ± 1.43	96.54 ± 0.65	96.92 ± 0.75	98.15 ± 0.52	8.33 ± 0.00	72.44 ± 0.00	78.21 ± 0.00	27.28 ± 0.00
darmanis	92.34 ± 3.14	77.02 ± 3.53	83.62 ± 2.82	85.11 ± 2.52	86.38 ± 2.55	86.60 ± 2.90	64.62 ± 4.10	8.51 ± 0.00	27.66 ± 0.00	6.38 ± 0.00	47.85 ± 0.00
lake	94.22 ± 0.68	78.85 ± 2.46	87.26 ± 0.56	84.60 ± 2.12	91.43 ± 0.90	72.13 ± 0.92	90.21 ± 0.67	23.33 ± 0.00	92.93 ± 0.00	78.82 ± 0.00	nan ± nan
Segerstolpe	99.16 ± 0.39	95.74 ± 1.06	96.91 ± 0.99	96.30 ± 0.50	98.45 ± 0.32	96.53 ± 0.65	97.33 ± 0.83	7.72 ± 0.00	90.40 ± 0.00	96.25 ± 0.00	92.78 ± 0.00
Tosches _turtle	96.44 ± 0.22	92.99 ± 0.12	94.45 ± 0.29	94.97 ± 0.24	94.80 ± 0.46	78.57 ± 4.56	96.45 ± 0.20	34.34 ± 0.00	96.79 ± 0.00	nan ± nan	nan ± nan
Xin	100.00 ± 0.00	99.17 ± 0.47	95.93 ± 1.42	99.72 ± 0.26	100.00 ± 0.00	93.03 ± 3.51	99.93 ± 0.14	40.00 ± 0.00	90.00 ± 0.00	100.00 ± 0.00	99.17 ± 0.00
Young	97.50 ± 0.60	88.94 ± 5.72	95.02 ± 0.89	92.37 ± 1.27	89.20 ± 0.69	85.19 ± 3.24	94.85 ± 0.51	25.37 ± 0.00	97.44 ± 0.00	87.25 ± 0.00	nan ± nan
Zheng 68K	77.55 ± 0.19	73.30 ± 0.76	72.75 ± 0.15	53.93 ± 0.80	59.41 ± 0.28	34.37 ± 4.48	59.47 ± 3.22	30.33 ± 0.00	76.56 ± 0.00	56.22 ± 0.00	27.31 ± 0.00
zillionis	97.80 ± 0.12	95.71 ± 0.19	96.80 ± 0.22	95.66 ± 0.16	95.08 ± 0.24	86.18 ± 2.39	97.46 ± 0.09	37.48 ± 0.00	98.43 ± 0.00	97.42 ± 0.00	nan ± nan
Adam	97.60 ± 0.58	96.78 ± 0.37	96.34 ± 0.80	83.11 ± 1.00	91.67 ± 0.73	94.84 ± 0.90	96.34 ± 0.64	nan ± nan	nan ± nan	31.15 ± 0.00	58.22 ± 0.00
AMB	87.78 ± 0.79	47.32 ± 1.30	91.69 ± 0.69	88.14 ± 0.63	91.64 ± 0.21	44.32 ± 3.20	85.07 ± 1.74	nan ± nan	nan ± nan	82.39 ± 0.00	nan ± nan
Bach	99.08 ± 0.15	96.63 ± 1.04	97.87 ± 0.23	97.81 ± 0.48	98.18 ± 0.16	90.24 ± 4.70	98.89 ± 0.24	nan ± nan	nan ± nan	98.77 ± 0.00	nan ± nan
Deng	99.27 ± 1.00	78.52 ± 2.51	86.30 ± 3.01	94.81 ± 4.44	88.52 ± 1.39	93.33 ± 1.48	89.26 ± 3.95	nan ± nan	nan ± nan	nan ± nan	nan ± nan
Baron Mouse	99.05 ± 0.71	94.07 ± 1.39	96.72 ± 0.40	97.30 ± 0.95	97.67 ± 0.35	95.98 ± 0.79	98.15 ± 0.53	nan ± nan	nan ± nan	94.97 ± 0.00	78.84 ± 0.00
usoskin	99.68 ± 0.44	97.44 ± 1.18	98.72 ± 1.09	91.20 ± 1.82	97.92 ± 1.65	98.72 ± 0.82	98.56 ± 0.60	nan ± nan	nan ± nan	15.20 ± 0.00	nan ± nan
Muraro	98.02 ± 0.27	96.47 ± 0.67	96.18 ± 0.87	95.01 ± 1.15	96.75 ± 0.58	96.75 ± 0.67	97.74 ± 0.77	nan ± nan	nan ± nan	nan ± nan	nan ± nan
campbell	90.45 ± 0.52	63.90 ± 2.11	79.15 ± 0.40	83.80 ± 0.37	89.74 ± 0.23	52.65 ± 2.57	88.72 ± 0.34	nan ± nan	nan ± nan	97.42 ± 0.00	nan ± nan
TM	96.65 ± 0.12	75.69 ± 2.50	96.46 ± 0.14	96.57 ± 0.12	89.93 ± 0.22	67.71 ± 4.38	94.15 ± 0.65	nan ± nan	nan ± nan	96.56 ± 0.00	nan ± nan
Klein	99.34 ± 0.46	99.34 ± 0.36	99.74 ± 0.28	92.35 ± 1.63	95.40 ± 0.82	98.24 ± 0.70	99.34 ± 0.28	nan ± nan	nan ± nan	97.24 ± 0.00	nan ± nan

F1-score, NMI and ARI score where we computed their mean value and its standard deviation according to their results. And the D2 and D3 shows their results. We also put the results of scGraphformer under accuracy metric on the 42 experiments and each five results for each experiment (Table D8). In this table, we perform our model in this experiment for two settings (set the training proportion of reference data for 0.8 and 1.0, respectively representing realistic and ideal situations while performing cross-platform experiments). The results in Table D8 shows that ideal performance is better that the realistic one. But we finally choose to set training proportion of reference dataset to 0.8. Regarding the use of 80% rather than 100% of the reference dataset, we expect to mirror real-world scenarios where researchers often set aside a portion of their data for validation. This practice helps ensure model generalizability and prevents overfitting. In our evaluation, we split the reference dataset into 80% for training and 20% for validation. While using 100% of the data for training is possible, it may not always reflect best practices in model development and validation. We set the same training proportion on all comparing methods while evaluating them on cross-platforms experiments. Therefore, we choose the 0.8-proportion as our results on the paper since the demands of realistic situation. Table D9 shows the results of each method while the training on SMART-seq2 and using the trained model to annotate 10Xv3. And the results correspond to the UMAP visualization in Figure 2e

Table C3 Comparison of methods on intra-experiments using F1-score.

method	scGraph -former	scVI	Cell -Typist	ACTINN	scmap -cell	scmap -cluster	scBal -ance	scBert (ZS)	scBert (FT)	scType	TOSICA
Baron Human	98.95 ± 0.25	95.13 ± 0.83	97.13 ± 0.40	92.27 ± 2.64	97.75 ± 0.19	97.37 ± 0.28	98.37 ± 0.19	20.12 ± 0.00	90.26 ± 0.97	94.03 ± 0.00	0.97 ± 0.00
campLiver	99.23 ± 0.84	90.17 ± 5.66	98.07 ± 1.16	96.90 ± 0.76	93.49 ± 1.43	96.51 ± 0.67	98.14 ± 0.53	1.29 ± 0.00	71.51 ± 8.75	23.92 ± 0.00	0.77 ± 0.00
darmanis	92.34 ± 3.14	69.43 ± 4.12	79.71 ± 2.56	85.02 ± 3.52	83.61 ± 3.14	85.29 ± 2.73	63.76 ± 5.96	2.00 ± 0.00	11.99 ± 0.00	34.37 ± 0.00	0.01 ± 0.00
lake	94.22 ± 0.68	73.38 ± 3.58	86.75 ± 0.63	64.79 ± 2.24	84.16 ± 2.28	91.57 ± 0.88	90.25 ± 0.62	18.06 ± 0.00	91.90 ± 0.44	nan ± nan	0.80 ± 0.00
Segerstolpe	99.16 ± 0.39	93.88 ± 1.46	95.81 ± 1.24	95.38 ± 0.94	96.39 ± 0.64	98.25 ± 0.41	97.02 ± 0.83	1.11 ± 0.00	85.40 ± 1.12	91.99 ± 0.00	0.96 ± 0.00
Tosches_turtle	96.44 ± 0.22	nan ± nan	94.35 ± 0.30	74.15 ± 6.00	94.84 ± 0.25	94.73 ± 0.47	96.44 ± 0.21	17.56 ± 0.00	96.75 ± 0.02	nan ± nan	nan ± nan
Xin	100.00 ± 0.00	99.16 ± 0.47	95.39 ± 1.75	90.24 ± 5.30	99.72 ± 0.26	100.00 ± 0.00	99.93 ± 0.14	32.68 ± 0.00	86.15 ± 0.00	99.16 ± 0.00	1.00 ± 0.00
Young	97.50 ± 0.60	nan ± nan	94.96 ± 0.91	82.03 ± 4.55	92.44 ± 1.22	89.36 ± 0.69	94.85 ± 0.52	15.34 ± 0.00	97.38 ± 0.04	nan ± nan	nan ± nan
Zheng 68K	77.55 ± 0.19	69.86 ± 1.07	72.15 ± 0.16	19.20 ± 5.70	54.27 ± 0.63	62.58 ± 0.22	61.96 ± 2.57	14.12 ± 0.00	75.89 ± 0.83	32.36 ± 0.00	0.59 ± 0.00
zillionis	97.80 ± 0.12	94.10 ± 0.21	96.72 ± 0.22	81.66 ± 3.15	95.06 ± 0.18	95.33 ± 0.20	97.51 ± 0.08	20.43 ± 0.00	98.32 ± 0.06	nan ± nan	0.97 ± 0.00
Adam	97.60 ± 0.58	96.77 ± 0.39	96.31 ± 0.81	94.77 ± 0.93	82.46 ± 1.28	91.81 ± 0.70	96.34 ± 0.65	nan ± nan	nan ± nan	58.07 ± 0.00	0.22 ± 0.00
AMB	87.78 ± 0.79	nan ± nan	91.34 ± 0.80	32.25 ± 3.51	87.76 ± 0.74	91.88 ± 0.20	85.29 ± 1.61	nan ± nan	nan ± nan	nan ± nan	0.83 ± 0.00
Bach	99.08 ± 0.15	95.29 ± 0.26	97.80 ± 0.26	86.77 ± 5.63	97.85 ± 0.46	98.22 ± 0.15	98.89 ± 0.23	nan ± nan	nan ± nan	nan ± nan	nan ± nan
Deng	99.27 ± 1.00	73.79 ± 4.14	86.32 ± 2.53	93.37 ± 1.18	94.77 ± 4.57	87.12 ± 2.21	88.33 ± 5.08	nan ± nan	nan ± nan	nan ± nan	nan ± nan
Baron Mouse	99.05 ± 0.71	92.05 ± 2.00	96.10 ± 0.70	94.75 ± 1.26	97.20 ± 1.02	97.67 ± 0.31	98.14 ± 0.53	nan ± nan	nan ± nan	83.50 ± 0.00	0.95 ± 0.00
usoskin	99.68 ± 0.44	97.36 ± 1.26	98.71 ± 1.10	98.70 ± 0.84	91.13 ± 1.76	97.83 ± 1.76	98.55 ± 0.60	nan ± nan	nan ± nan	nan ± nan	0.04 ± 0.00
Muraro	98.02 ± 0.27	95.91 ± 0.91	96.16 ± 0.89	96.72 ± 0.70	94.82 ± 1.32	96.78 ± 0.57	97.75 ± 0.76	nan ± nan	nan ± nan	nan ± nan	nan ± nan
campbell	90.45 ± 0.52	55.53 ± 2.07	78.09 ± 0.48	44.58 ± 2.55	83.18 ± 0.45	89.87 ± 0.23	88.55 ± 0.31	nan ± nan	nan ± nan	nan ± nan	0.85 ± 0.00
TM	96.65 ± 0.12	67.86 ± 3.00	96.25 ± 0.19	58.77 ± 5.56	96.45 ± 0.13	90.53 ± 0.20	94.62 ± 0.37	nan ± nan	nan ± nan	nan ± nan	0.97 ± 0.00
Klein	99.34 ± 0.46	99.33 ± 0.37	99.74 ± 0.28	98.21 ± 0.73	92.61 ± 1.60	95.48 ± 0.80	99.33 ± 0.28	nan ± nan	nan ± nan	nan ± nan	0.97 ± 0.00

in manuscript. For summarizing results on all experiments, we computed the mean, median and standard variation values of scGraphformer and other comparison methods across all 42 experiments which is shown in Table D10.

Meanwhile, we trained our model using datasets fused by multiple sequencing technologies, excluding the query datasets for testing in each fused dataset. Specifically, in our cross-platform experiments, we evaluated our model on PBMC datasets sampled using seven different technologies: Seq-Well, inDrop-seq, CEL-Seq, 10Xv2, Drop-Seq, Smart-Seq2, and 10Xv3. To clarify, when evaluating one of the sequencing datasets, we integrated the remaining six datasets into a single fusion dataset. This integration was done in such a way that no genomic information was lost; genes not present in the original data were represented with zero expression in the fused dataset. And it resulted in seven distinct fused datasets. Each fused dataset was then used as a reference, while the dataset excluded from the fusion served as the query dataset. The model was trained on the reference dataset and subsequently used to annotate the query dataset. The results regarding to the Figure 2b in manuscript is shown in Table D11. Meanwhile, we computed the average values of F1-score, ARI, and Cohen's Kappa Score across all experiments and the result are shown in Table D12.

Table C4 Comparison of methods on intra-experiments using Cohen's Kappa Score.

method	scGraph-former	scVI	Cell-Typist	scmap-cell	scmap-cluster	scBal-ance	ACTINN	scBert (ZS)	scBert (FT)	scType	TOSICA
Baron Human	98.40 ± 0.35	95.35 ± 0.69	96.63 ± 0.42	97.27 ± 0.21	96.74 ± 0.32	97.99 ± 0.24	92.17 ± 2.31	0.18 ± 0.00	88.78 ± 0.97	92.73 ± 0.00	0.97 ± 0.00
campLiver	99.08 ± 1.00	90.96 ± 4.56	97.68 ± 1.40	92.20 ± 1.69	95.83 ± 0.81	97.78 ± 0.63	96.29 ± 0.94	0.00 ± 0.00	69.68 ± 8.70	20.97 ± 0.00	0.74 ± 0.00
darmanis	90.59 ± 3.87	71.83 ± 6.44	79.62 ± 3.55	81.37 ± 3.13	83.15 ± 3.33	57.59 ± 5.02	83.24 ± 3.79	0.76 ± 0.00	0.00 ± 0.00	34.52 ± 0.00	0.02 ± 0.00
lake	91.13 ± 0.80	71.15 ± 4.60	84.38 ± 0.79	81.60 ± 2.65	89.86 ± 1.01	88.36 ± 0.87	64.96 ± 1.60	6.73 ± 0.00	90.60 ± 0.48	nan ± nan	0.75 ± 0.00
Segerstolpe	98.60 ± 0.55	94.42 ± 1.36	95.95 ± 1.29	95.19 ± 0.66	97.99 ± 0.41	96.52 ± 1.07	95.47 ± 0.80	0.00 ± 0.00	85.11 ± 1.02	90.59 ± 0.00	0.95 ± 0.00
Tosches_turtle	95.69 ± 0.28	nan ± nan	93.23 ± 0.35	93.89 ± 0.28	93.70 ± 0.55	95.70 ± 0.24	73.58 ± 5.75	0.00 ± 0.00	96.12 ± 0.03	nan ± nan	nan ± nan
Xin	100.00 ± 0.00	98.49 ± 0.83	92.27 ± 2.67	99.49 ± 0.48	100.00 ± 0.00	99.87 ± 0.27	86.53 ± 6.70	0.40 ± 0.00	79.77 ± 0.00	98.47 ± 0.00	1.00 ± 0.00
Young	97.04 ± 0.72	nan ± nan	94.05 ± 1.07	90.93 ± 1.47	87.26 ± 0.82	93.86 ± 0.61	82.05 ± 3.97	1.92 ± 0.00	96.89 ± 0.05	nan ± nan	nan ± nan
Zheng 68K	69.98 ± 0.36	66.31 ± 0.66	65.21 ± 0.24	42.78 ± 0.77	52.42 ± 0.27	52.52 ± 2.94	6.38 ± 7.75	0.00 ± 0.00	70.25 ± 1.14	21.74 ± 0.00	0.49 ± 0.00
zillionis	97.07 ± 0.16	94.23 ± 0.27	95.73 ± 0.30	94.21 ± 0.20	93.55 ± 0.32	96.65 ± 0.11	81.07 ± 3.34	0.00 ± 0.00	97.82 ± 0.08	nan ± nan	0.97 ± 0.00
Adam	97.21 ± 0.67	96.26 ± 0.44	95.75 ± 0.93	80.30 ± 1.21	90.32 ± 0.85	95.76 ± 0.74	94.00 ± 1.05	nan ± nan	nan ± nan	53.85 ± 0.00	0.23 ± 0.00
AMB	82.37 ± 0.91	nan ± nan	91.40 ± 0.72	87.72 ± 0.65	91.37 ± 0.21	84.58 ± 1.78	41.61 ± 3.54	nan ± nan	nan ± nan	nan ± nan	0.82 ± 0.00
Bach	98.64 ± 0.20	95.68 ± 1.33	97.28 ± 0.29	97.22 ± 0.60	97.69 ± 0.20	98.58 ± 0.30	87.31 ± 6.27	nan ± nan	nan ± nan	nan ± nan	0.98 ± 0.00
Deng	97.47 ± 2.47	69.18 ± 3.45	80.46 ± 4.04	92.59 ± 6.28	83.20 ± 2.23	84.41 ± 5.74	90.39 ± 2.06	nan ± nan	nan ± nan	nan ± nan	nan ± nan
Baron Mouse	98.67 ± 1.00	91.73 ± 1.95	95.40 ± 0.59	96.23 ± 1.36	96.79 ± 0.47	97.44 ± 0.72	94.40 ± 1.10	nan ± nan	nan ± nan	73.28 ± 0.00	0.93 ± 0.00
usoskin	98.66 ± 0.93	96.44 ± 1.67	98.25 ± 1.48	87.88 ± 2.55	97.12 ± 2.27	98.02 ± 0.80	98.24 ± 1.12	nan ± nan	nan ± nan	nan ± nan	0.00 ± 0.00
Muraro	97.07 ± 0.47	95.47 ± 0.85	95.07 ± 1.08	93.49 ± 1.48	95.81 ± 0.72	97.08 ± 0.97	95.77 ± 0.89	nan ± nan	nan ± nan	nan ± nan	nan ± nan
campbell	87.91 ± 0.84	56.14 ± 2.48	73.34 ± 0.59	80.61 ± 0.44	87.81 ± 0.31	86.52 ± 0.41	41.45 ± 3.36	nan ± nan	nan ± nan	nan ± nan	0.82 ± 0.00
TM	95.04 ± 0.24	73.55 ± 2.76	96.18 ± 0.15	96.31 ± 0.13	89.19 ± 0.23	93.72 ± 0.70	64.30 ± 5.04	nan ± nan	nan ± nan	nan ± nan	0.96 ± 0.00
Klein	99.08 ± 0.64	99.08 ± 0.51	99.64 ± 0.38	89.51 ± 2.19	93.65 ± 1.10	99.08 ± 0.38	97.54 ± 0.98	nan ± nan	nan ± nan	nan ± nan	0.96 ± 0.00

Appendix E Mouse brain dataset.

Details of while training on Zeisel and annotate Rosenberg is shown in E13 where we use accuracy score as metric.

Appendix F Large-scale datasets

While using Zheng 68K dataset, our results using F1-score as metric is shown in F14. The Covid-19 atlas is training using all of its sub-celltypes and the cell type distribution is shown in fig F4. Each cell concludes its sub-cell types which are more similarity than others. While training, we used sub-cell types as training labels and there are 64 cell types in total.

Appendix G Influence of KNN graph

It is possible to augment scGraphformer with existing high-quality cell graphs. In this study, we opt for a K-nearest neighbors (KNN) graph, a common choice in single-cell analyses. It is important to note that the incorporation of such a graph into scGraphformer is not mandatory; our findings in fig G5 demonstrate that the integration of existing, potentially noisy, cell graphs does not invariably lead to enhanced performance. While not adding the relational bias to scGraphformer, the all-pair cell network

Table C5 scGraphformer's performance on intra-datasets for five-time experiments. In each independent repeating experiment, the training, validation and testing sets were randomly selected under the same splitting proportion. We perform the same settings for each method.

dataset	accuracy[0]	accuracy[1]	accuracy[2]	accuracy[3]	accuracy[4]
Deng	98.18	100	100	100	98.18
Xin	100	100	100	100	100
darmanis	95.74	88.3	90.43	93.62	92.55
Baron Mouse	98.68	99.47	99.74	98.15	99.74
Segerstolpe	98.83	99.3	98.83	99.07	99.77
campLiver	99.36	100	98.72	100	98.72
usoskin	99.2	100	100	99.2	100
AMB	88.43	88.16	88.24	86.48	87.57
lake	94.75	93.1	94.75	94.09	94.42
Baron Human	99.01	98.83	98.72	99.36	98.83
Muraro	97.65	98.12	98.12	98.35	97.88
Zheng 68K	77.25	77.49	77.75	77.57	77.68
zillionis	97.63	97.6	97.48	97.64	97.67
campbell	90.56	90.78	90.04	89.81	91.06
TM	96.61	96.61	96.76	96.49	96.8
Tosches_turtle	96.09	96.87	96.52	96.68	95.9
Young	97.27	97.63	97.36	97.63	97.71
Klein	98.71	98.9	98.9	98.9	98.71
Bach	98.94	99.33	99.09	98.99	99.05
Adam	98.22	97.4	97.95	97.95	96.99

Table C6 Comparison of methods on each cell type of Baron Human. We stored the annotation results of each method on each experiment and we then used the annotation results to compute the accuracy of each cell type. Baron human dataset is a classical imbalanced dataset where minor cell types exist. And scGraphformer outperforms most of the cell types. We have stored all of the annotation results on all experiments and we show the table of Baron Human as an example.

method	scGraph -former	Cell -Typist	scBalance	scmap -cell	scVI	ACTINN	scmap -cluster	TOSICA	scBert	scType
acinar	0.9969 ± 0.0000	0.9655 ± 0.0060	0.9825 ± 0.0066	0.9951 ± 0.0000	0.9892 ± 0.0041	0.9764 ± 0.0022	0.9951 ± 0.0000	0.0000 ± 0.0000	0.9477 ± 0.0163	0.9906 ± 0.0000
beta	0.9980 ± 0.0000	0.9980 ± 0.0000	0.9952 ± 0.0011	0.9980 ± 0.0000	0.9941 ± 0.0020	0.9965 ± 0.0016	0.9980 ± 0.0000	0.9804 ± 0.0100	0.9586 ± 0.0194	0.9778 ± 0.0000
delta	0.9884 ± 0.0000	0.9254 ± 0.0174	0.9851 ± 0.0030	0.9661 ± 0.0000	0.9881 ± 0.0046	0.8271 ± 0.1451	0.9661 ± 0.0000	0.9831 ± 0.0100	0.7081 ± 0.2922	0.9484 ± 0.0000
activated _stellate	0.9824 ± 0.0000	0.9560 ± 0.0167	0.9545 ± 0.0000	0.9600 ± 0.0000	0.9520 ± 0.0268	0.9120 ± 0.0610	0.9600 ± 0.0000	0.0000 ± 0.0000	0.9313 ± 0.0348	0.0000 ± 0.0000
ductal	0.9870 ± 0.0000	0.9960 ± 0.0042	0.9976 ± 0.0036	0.9747 ± 0.0000	0.9869 ± 0.0045	0.9747 ± 0.0101	0.9747 ± 0.0000	0.9949 ± 0.0100	0.9037 ± 0.0223	0.8310 ± 0.0000
alpha	0.9987 ± 0.0000	0.9991 ± 0.0012	0.9912 ± 0.0019	0.9936 ± 0.0000	0.9957 ± 0.0015	0.9940 ± 0.0041	0.9936 ± 0.0000	0.9808 ± 0.0100	0.9821 ± 0.0268	0.9875 ± 0.0000
epsilon	0.8333 ± 0.0000	0.0000 ± 0.0000	0.9750 ± 0.0559	0.7143 ± 0.0000	0.0000 ± 0.0000	0.0000 ± 0.0000	0.7143 ± 0.0000	1.0000 ± 0.0100	0.0000 ± 0.0000	0.0000 ± 0.0000
gamma	0.9686 ± 0.0000	0.8367 ± 0.0144	1.0000 ± 0.0000	0.8980 ± 0.0000	0.9918 ± 0.0183	0.1551 ± 0.2143	0.8980 ± 0.0000	1.0000 ± 0.0100	0.5349 ± 0.1274	0.0000 ± 0.0000
endothelial	1.0000 ± 0.0000	0.9800 ± 0.0075	0.9676 ± 0.0121	0.9167 ± 0.0000	0.9533 ± 0.0217	0.8900 ± 0.0253	0.9167 ± 0.0000	0.9333 ± 0.0100	0.8045 ± 0.0111	0.9802 ± 0.0000
quiescent _stellate	0.9769 ± 0.0000	0.8867 ± 0.0183	0.8667 ± 0.0215	0.8333 ± 0.0000	0.4933 ± 0.4506	0.7533 ± 0.0989	0.8333 ± 0.0000	0.8000 ± 0.0100	0.1758 ± 0.1439	0.0000 ± 0.0000
macrophage	1.0000 ± 0.0000	1.0000 ± 0.0000	0.9667 ± 0.0456	1.0000 ± 0.0000	0.2000 ± 0.4472	0.1333 ± 0.2981	1.0000 ± 0.0000	1.0000 ± 0.0100	0.6000 ± 0.1333	0.0000 ± 0.0000
schwann	1.0000 ± 0.0000	0.6400 ± 0.0894	0.9333 ± 0.0913	0.8000 ± 0.0000	0.0000 ± 0.0000	0.0000 ± 0.0000	0.8000 ± 0.0000	0.8000 ± 0.0100	0.0000 ± 0.0000	0.0000 ± 0.0000
mast	1.0000 ± 0.0000	1.0000 ± 0.0000	1.0000 ± 0.0000	1.0000 ± 0.0000	0.2000 ± 0.4472	0.0000 ± 0.0000	1.0000 ± 0.0000	1.0000 ± 0.0100	0.0000 ± 0.0000	0.0000 ± 0.0000
t_cell	1.0000 ± 0.0000	0.4000 ± 0.3651	1.0000 ± 0.0000	1.0000 ± 0.0000	0.0000 ± 0.0000	0.0000 ± 0.0000	1.0000 ± 0.0000	0.3333 ± 0.0100	0.0000 ± 0.0000	0.0000 ± 0.0000

Table D7 Comparison of methods on inter-datasets on Accuracy score. There are 42 experiments where each one was evaluated for five times. And values represent mean accuracy and standard deviation over the five results.

method	scGraphformer	scVI	CellTypist	scBalance	ACTINN	scmap -cluster	scmap-cell
Seq-Well_inDrop	93.44 ± 0.31	93.09 ± 0.04	91.92 ± 0.02	92.44 ± 0.08	83.02 ± 6.81	85.65 ± 0.00	89.19 ± 0.00
Seq-Well_CEL-Seq	90.16 ± 0.36	90.45 ± 0.01	88.68 ± 0.01	89.65 ± 0.08	78.92 ± 5.58	81.97 ± 0.00	86.56 ± 0.00
Seq-Well_10Xv2	89.27 ± 0.32	87.76 ± 0.08	87.66 ± 0.06	88.64 ± 0.16	79.84 ± 3.79	82.53 ± 0.00	85.60 ± 0.00
Seq-Well_Drop-Seq	88.94 ± 0.25	88.00 ± 0.16	86.82 ± 0.05	87.94 ± 0.22	79.15 ± 5.63	80.95 ± 0.00	85.45 ± 0.00
Seq-Well_Smart-Seq2	94.30 ± 0.36	94.45 ± 0.04	92.55 ± 0.03	93.46 ± 0.14	82.99 ± 4.31	85.69 ± 0.00	90.39 ± 0.00
Seq-Well_10Xv3	90.44 ± 0.53	89.62 ± 0.05	88.80 ± 0.03	89.73 ± 0.11	76.52 ± 5.51	82.12 ± 0.00	86.68 ± 0.00
inDrop_Seq-Well	94.94 ± 0.54	95.75 ± 0.20	93.87 ± 0.04	95.21 ± 0.07	86.26 ± 3.04	87.06 ± 0.00	91.74 ± 0.00
inDrop_CEL-Seq	92.54 ± 0.46	90.41 ± 0.04	91.00 ± 0.05	92.37 ± 0.09	79.84 ± 4.82	84.61 ± 0.00	89.14 ± 0.00
inDrop_10Xv2	90.48 ± 0.30	89.73 ± 1.41	88.95 ± 0.03	90.43 ± 0.10	77.15 ± 7.20	83.75 ± 0.00	87.10 ± 0.00
inDrop_Drop-Seq	90.46 ± 0.48	89.14 ± 1.22	88.59 ± 0.05	90.13 ± 0.17	79.58 ± 5.07	82.86 ± 0.00	87.45 ± 0.00
inDrop_Smart-Seq2	96.46 ± 0.43	96.11 ± 1.37	95.04 ± 0.03	96.41 ± 0.15	82.12 ± 4.49	88.51 ± 0.00	93.08 ± 0.00
inDrop_10Xv3	91.43 ± 0.23	91.19 ± 1.34	90.31 ± 0.02	91.57 ± 0.12	76.81 ± 5.70	83.58 ± 0.00	88.32 ± 0.00
CEL-Seq_Seq-Well	95.17 ± 0.36	95.66 ± 0.09	93.49 ± 0.05	94.14 ± 0.21	82.29 ± 13.02	81.17 ± 0.00	89.59 ± 0.00
CEL-Seq_inDrop	94.91 ± 0.68	92.96 ± 0.16	93.79 ± 0.03	94.32 ± 0.21	83.64 ± 2.93	83.24 ± 0.00	90.05 ± 0.00
CEL-Seq_10Xv2	93.72 ± 0.25	89.91 ± 1.71	91.64 ± 0.03	92.99 ± 0.17	76.28 ± 5.44	82.19 ± 0.00	88.85 ± 0.00
CEL-Seq_Drop-Seq	94.09 ± 0.42	89.23 ± 1.50	91.91 ± 0.04	93.32 ± 0.15	76.66 ± 7.60	81.96 ± 0.00	88.97 ± 0.00
CEL-Seq_Smart-Seq2	96.67 ± 0.42	94.50 ± 0.05	94.91 ± 0.05	95.90 ± 0.22	82.80 ± 4.71	83.02 ± 0.00	91.38 ± 0.00
CEL-Seq_10Xv3	95.70 ± 0.25	91.18 ± 1.80	93.53 ± 0.07	94.83 ± 0.36	79.29 ± 5.50	82.27 ± 0.00	90.16 ± 0.00
10Xv2_Seq-Well	96.11 ± 0.85	96.32 ± 0.48	94.10 ± 0.11	94.85 ± 0.25	79.59 ± 6.40	82.80 ± 0.00	90.59 ± 0.00
10Xv2_inDrop	95.18 ± 0.49	94.60 ± 1.46	93.72 ± 0.06	94.53 ± 0.17	82.85 ± 6.53	83.94 ± 0.00	89.59 ± 0.00
10Xv2_CEL-Seq	95.28 ± 1.26	92.91 ± 1.37	93.91 ± 0.03	95.03 ± 0.08	77.75 ± 12.56	83.22 ± 0.00	90.36 ± 0.00
10Xv2_Drop-Seq	94.15 ± 0.49	90.15 ± 1.83	92.23 ± 0.02	93.96 ± 0.19	79.92 ± 3.18	83.20 ± 0.00	89.66 ± 0.00
10Xv2_Smart-Seq2	95.95 ± 0.90	95.47 ± 1.41	94.21 ± 0.03	95.07 ± 0.32	83.87 ± 4.90	83.23 ± 0.00	90.94 ± 0.00
10Xv2_10Xv3	96.08 ± 0.56	92.27 ± 2.21	93.81 ± 0.05	95.02 ± 0.22	81.06 ± 3.26	83.23 ± 0.00	91.09 ± 0.00
Drop-Seq_Seq-Well	95.92 ± 0.46	95.69 ± 0.03	94.20 ± 0.06	94.84 ± 0.10	85.44 ± 3.87	81.56 ± 0.00	90.20 ± 0.00
Drop-Seq_inDrop	95.18 ± 0.51	93.17 ± 0.08	93.74 ± 0.02	94.44 ± 0.13	83.33 ± 7.46	82.98 ± 0.00	89.46 ± 0.00
Drop-Seq_CEL-Seq	95.55 ± 0.65	92.47 ± 2.61	94.18 ± 0.02	95.09 ± 0.26	83.62 ± 1.14	82.83 ± 0.00	90.07 ± 0.00
Drop-Seq_10Xv2	94.72 ± 0.77	88.99 ± 1.39	92.82 ± 0.02	94.13 ± 0.15	79.41 ± 5.07	83.57 ± 0.00	89.62 ± 0.00
Drop-Seq_Smart-Seq2	96.24 ± 1.05	94.39 ± 0.16	94.43 ± 0.03	94.97 ± 0.18	82.05 ± 7.92	82.25 ± 0.00	90.73 ± 0.00
Drop-Seq_10Xv3	95.00 ± 0.49	91.77 ± 1.74	93.88 ± 0.05	95.05 ± 0.21	75.72 ± 8.27	82.45 ± 0.00	90.38 ± 0.00
Smart-Seq2_Seq-Well	95.36 ± 0.38	95.46 ± 0.02	93.83 ± 0.03	94.54 ± 0.08	83.92 ± 7.87	86.76 ± 0.00	91.34 ± 0.00
Smart-Seq2_inDrop	95.53 ± 0.59	94.74 ± 1.33	94.05 ± 0.05	95.04 ± 0.10	81.10 ± 5.69	88.41 ± 0.00	92.22 ± 0.00
Smart-Seq2_CEL-Seq	92.92 ± 0.44	92.96 ± 0.04	91.38 ± 0.05	92.28 ± 0.09	80.86 ± 3.26	84.54 ± 0.00	88.98 ± 0.00
Smart-Seq2_10Xv2	90.22 ± 0.34	90.19 ± 0.05	88.48 ± 0.06	89.59 ± 0.12	80.61 ± 3.39	83.29 ± 0.00	86.58 ± 0.00
Smart-Seq2_Drop-Seq	90.23 ± 0.21	89.85 ± 1.01	88.58 ± 0.06	89.49 ± 0.16	80.78 ± 1.85	82.24 ± 0.00	86.97 ± 0.00
Smart-Seq2_10Xv3	91.82 ± 0.53	91.05 ± 1.24	90.59 ± 0.05	91.46 ± 0.05	78.95 ± 6.42	83.73 ± 0.00	88.30 ± 0.00
10Xv3_Seq-Well	96.30 ± 0.15	95.73 ± 0.26	93.74 ± 0.06	94.29 ± 0.27	82.16 ± 6.69	81.04 ± 0.00	89.83 ± 0.00
10Xv3_inDrop	95.26 ± 0.36	94.54 ± 1.35	93.35 ± 0.04	93.65 ± 0.14	82.94 ± 5.28	81.96 ± 0.00	88.92 ± 0.00
10Xv3_CEL-Seq	96.46 ± 0.56	92.91 ± 2.36	93.95 ± 0.04	95.25 ± 0.26	75.99 ± 6.38	81.87 ± 0.00	90.40 ± 0.00
10Xv3_10Xv2	94.71 ± 0.40	90.34 ± 2.36	92.23 ± 0.09	93.43 ± 0.26	80.82 ± 1.24	82.12 ± 0.00	89.28 ± 0.00
10Xv3_Drop-Seq	94.24 ± 0.55	90.49 ± 2.55	91.67 ± 0.09	93.04 ± 0.22	79.63 ± 3.29	81.41 ± 0.00	89.28 ± 0.00
10Xv3_Smart-Seq2	97.01 ± 0.28	95.29 ± 1.57	94.44 ± 0.06	95.06 ± 0.38	79.88 ± 10.72	82.06 ± 0.00	90.69 ± 0.00

Table D8 scGraphformer's five results on each inter-experiment. We perform our model for two settings, one of which is setting the training proportion of reference to 100% which is close to the ideal situation, and another one is setting the training proportion to 80% which is close to the realistic situation. We use the 80%-proportion for all comparison methods as our final evaluating performance.

	Training Proportion=1					Training Proportion=0.8				
dataset	acc[0]	acc[1]	acc[2]	acc[3]	acc[4]	acc[0]	acc[1]	acc[2]	acc[3]	acc[4]
Seq-Well_inDrop	94.03	94.01	94.07	94.12	93.96	92.75	92.98	92.48	92.54	92.39
Seq-Well_CEL-Seq	91.85	91.86	91.9	91.82	91.77	89.9	89.83	89.76	89.99	89.76
Seq-Well_10Xv2	89.78	89.77	89.75	89.83	89.73	88.56	88.7	88.58	88.44	88.67
Seq-Well_Drop-Seq	89.68	89.59	89.52	89.5	89.65	88.24	88.28	88.11	87.9	88.45
Seq-Well_Smart-Seq2	95.81	95.81	95.84	95.76	95.79	93.84	93.87	93.74	93.84	93.69
Seq-Well_10Xv3	91.71	91.58	91.63	91.57	91.59	89.8	89.96	89.71	89.79	89.8
inDrop_Seq-Well	96.79	96.86	96.68	96.51	96.69	95.19	94.77	94.99	94.98	94.91
inDrop_CEL-Seq	94.14	94.2	94.24	94.39	94.29	92.22	91.93	92.03	92.18	91.97
inDrop_10Xv2	91.41	91.6	91.38	91.6	91.47	90.08	89.94	89.8	90.21	90.05
inDrop_Drop-Seq	91.81	91.85	91.71	91.65	91.62	90.14	90.12	89.76	90.23	90.08
inDrop_Smart-Seq2	98.46	98.73	98.44	98.4	98.58	96.4	95.9	96.15	96.33	96.12
inDrop_10Xv3	93.44	93.45	93.32	93.51	93.63	91.45	91.19	91.27	91.45	91.04
CEL-Seq_Seq-Well	97.09	97.17	97.02	97.21	96.92	94.41	94.32	94.4	94.34	94.54
CEL-Seq_inDrop	96.92	96.76	96.82	96.93	96.83	94.29	94.65	94.36	94.26	94.58
CEL-Seq_10Xv2	95.43	95.41	95.63	95.59	95.61	92.97	92.93	92.88	92.41	92.8
CEL-Seq_Drop-Seq	95.69	95.64	95.81	95.78	95.91	93.42	93.39	93.11	93.03	92.89
CEL-Seq_Smart-Seq2	98.48	98.71	98.71	98.71	98.84	96.07	96.02	95.97	95.97	95.9
CEL-Seq_10Xv3	97.68	97.55	97.85	97.46	97.63	94.62	94.86	94.5	94.41	94.64
10Xv2_Seq-Well	97.38	96.94	97.26	96.95	97.12	94.79	95.18	95.09	95.39	95.38
10Xv2_inDrop	96.74	96.72	96.68	96.62	96.56	94.21	94.65	94.78	94.6	94.51
10Xv2_CEL-Seq	97.88	97.85	98.14	98.11	98.28	95.08	95.53	95.14	95.33	95.46
10Xv2_Drop-Seq	95.84	95.63	95.77	96.03	95.79	93.85	94.06	93.81	93.98	93.6
10Xv2_Smart-Seq2	98.6	98.53	98.69	98.41	98.2	95.5	96.01	95.84	95.9	95.86
10Xv2_10Xv3	97.58	97.19	97.37	97.27	97.58	94.99	95.56	94.9	95.3	95.29
Drop-Seq_Seq-Well	96.73	97.03	96.93	96.83	96.91	95.06	94.99	95.22	95.06	94.89
Drop-Seq_inDrop	96.62	96.58	96.65	96.5	96.59	94.43	94.67	94.62	94.35	94.5
Drop-Seq_CEL-Seq	98.19	98.25	98.13	98.14	97.99	95.05	95.46	95.48	95.49	95.54
Drop-Seq_10Xv2	95.49	95.34	95.53	95.47	95.44	93.77	93.97	93.7	94.06	93.59
Drop-Seq_Smart-Seq2	98.35	98.32	98.34	98.7	98.17	95.85	95.87	95.9	95.99	96.04
Drop-Seq_10Xv3	97.22	97.61	97.71	97.37	97.2	95.02	95.24	95.07	95	95.34
Smart-Seq2_Seq-Well	96.94	97.04	96.94	97.01	97.15	94.96	94.61	94.71	94.64	94.91
Smart-Seq2_inDrop	97.06	96.9	96.89	97.02	97.07	95.14	94.89	94.87	94.9	95.22
Smart-Seq2_CEL-Seq	94.45	94.44	94.46	94.33	94.74	92.14	92.3	92.17	92.27	92.05
Smart-Seq2_10Xv2	91.5	91.54	91.59	91.46	91.65	89.75	89.68	89.35	89.43	89.6
Smart-Seq2_Drop-Seq	91.57	91.72	91.79	91.52	91.74	89.99	89.71	89.56	89.79	89.76
Smart-Seq2_10Xv3	93.85	93.84	93.73	93.62	93.61	91.53	91.3	91.16	91.48	91.41
10Xv3_Seq-Well	97.52	97.41	97.38	97.46	97.52	94.85	94.75	95.14	95.28	95.56
10Xv3_inDrop	96.64	96.81	96.81	96.97	96.85	94.26	94.23	94.34	94.29	94.49
10Xv3_CEL-Seq	98.42	98.38	98.31	98.38	98.44	95.17	94.96	95.14	95.07	95.47
10Xv3_10Xv2	95.98	95.74	95.62	95.99	95.94	93.97	93.35	93.94	93.73	93.6
10Xv3_Drop-Seq	95.87	95.61	95.52	96.14	95.89	93.12	93.12	93.61	93.38	93.78
10Xv3_Smart-Seq2	98.85	98.8	98.61	98.49	98.71	95.87	95.81	96.03	95.94	95.97

Table D9 Comparison of each methods while training on Smart-seq2 and testing on 10Xv3

CellType	scGraphformer	ACTINN	CellTypist	scBalance	scmap-cell	scmap -cluster	scVI
CD4+ T cell	94.48	73.49	91.6	93.79	86.62	83.69	93.99
Cytotoxic T cell	96.55	93.27	95.9	95.94	91.74	87.07	95.35
Dendritic cell	0	0	0	0	0	0	0
B cell	99.13	94.86	97.87	99.83	98	97.3	99.35
CD14+ monocyte	98.1	95.73	98.71	98.84	93.97	87.41	99.55
Natural killer cell	0	0	0	0	0	0	0
Megakaryocyte	99.34	97.46	99.23	100	98.67	98.45	99.89
CD16+ monocyte	97.32	0	91.95	98.39	97.5	96.24	0

Table D10 Summarizing values comparison across all 42 cross-experiments.

	Accuracy			F1-Score			NMI			Cohen's Kappa		
method	median	mean	std	median	mean	std	median	mean	std	median	mean	std
scGraphformer	94.92	94.01	2.31	94.29	92.83	3.48	86.8	86.71	2.24	93.05	91.77	2.98
scBalance	94.13	93.28	2.19	93.96	92.43	3.42	85.84	85.75	1.97	92.37	91.25	2.84
scVI	92.69	92.4	2.52	92.94	91.35	3.41	86.02	85.86	2.62	90.38	90.04	3.23
CellTypist	93.42	92.26	2.26	90.6	90.31	3.61	83.47	83.03	1.9	91.29	89.88	2.92
scmap-cell	89.61	89.41	1.73	90.07	89	3.01	78.04	77.86	1.58	86.63	86.28	2.23
scmap -cluster	82.92	83.28	1.81	83.78	83.57	2.44	69.64	69.76	1.57	78.27	78.56	2.17
ACTINN	80.69	80.61	2.64	76.69	76.74	3.74	69.21	69.64	1.93	74.46	73.95	3.48

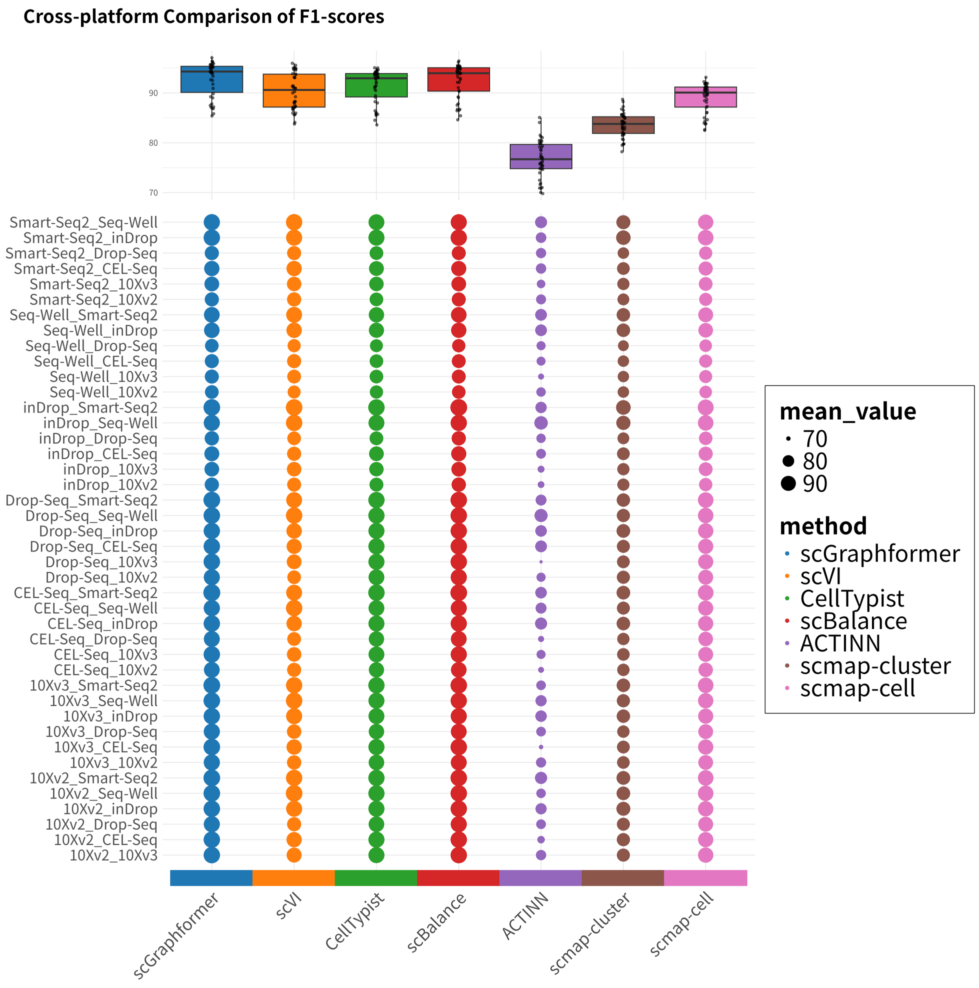

Fig. D2 Comparison on cross-platform experiments using F1-Score metric.

Table D11 Evaluation on fused dataset using Accuracy score.

Reference	merge_except_10Xv3	merge_except_10Xv2	merge_except_Seq-Well	merge_except_Smart-Seq2	merge_except_CEL-Seq	merge_except_Drop-Seq	merge_except_inDrop
Query	10Xv3	10Xv2	Seq-Well	Smart-Seq2	CEL-Seq	Drop-Seq	inDrop
MeanAccuracy	97.82	95.89	98.00	99.07	98.24	95.27	97.12
StdAccuracy	0.34	0.47	0.34	0.06	0.13	0.53	0.08
accuracy[0]	97.50	96.05	97.64	99.08	98.20	95.39	97.03
accuracy[1]	98.01	96.52	98.44	99.04	98.24	95.94	97.06
accuracy[2]	97.41	95.84	98.02	99.12	98.45	95.56	97.13
accuracy[3]	98.14	95.84	98.23	98.99	98.23	94.83	97.25
accuracy[4]	98.05	95.21	97.69	99.13	98.09	94.64	97.12

Table D12 Evaluation on fused dataset using different metrics.

model	metric	Reference	Query	MeanValue	StdValue	Value0	Value1	Value2	Value3	Value4
scGraphformer	F1-Score	merge_except _10Xv3	10Xv3	97.93	0.24	98.19	97.66	97.96	98.13	97.70
scGraphformer	F1-Score	merge_except _10Xv2	10Xv2	95.96	0.22	95.96	96.02	96.04	96.18	95.60
scGraphformer	F1-Score	merge_except _Seq-Well	Seq-Well	97.66	0.16	97.73	97.40	97.82	97.74	97.63
scGraphformer	F1-Score	merge_except _Smart-Seq2	Smart-Seq2	98.89	0.12	98.97	98.84	98.99	98.95	98.71
scGraphformer	F1-Score	merge_except _CEL-Seq	CEL-Seq	98.30	0.10	98.33	98.14	98.40	98.24	98.37
scGraphformer	F1-Score	merge_except _Drop-Seq	Drop-Seq	96.05	0.28	96.32	95.86	96.34	95.98	95.73
scGraphformer	F1-Score	merge_except _inDrop	inDrop	97.10	0.10	97.23	97.14	97.14	97.02	96.97
scGraphformer	Cohen's Kappa	merge_except _10Xv3	10Xv3	97.26	0.37	96.87	97.45	96.85	97.63	97.51
scGraphformer	Cohen's Kappa	merge_except _10Xv2	10Xv2	95.04	0.39	95.35	95.57	94.76	94.74	94.77
scGraphformer	Cohen's Kappa	merge_except _Seq-Well	Seq-Well	97.33	0.37	97.04	97.75	97.46	97.53	96.86
scGraphformer	Cohen's Kappa	merge_except _Smart-Seq2	Smart-Seq2	98.74	0.07	98.73	98.70	98.77	98.65	98.84
scGraphformer	Cohen's Kappa	merge_except _CEL-Seq	CEL-Seq	97.75	0.16	97.72	97.74	98.01	97.74	97.57
scGraphformer	Cohen's Kappa	merge_except _Drop-Seq	Drop-Seq	94.44	0.36	94.51	95.02	94.33	94.27	94.06
scGraphformer	Cohen's Kappa	merge_except _inDrop	inDrop	96.47	0.07	96.45	96.47	96.56	96.38	96.51
scGraphformer	ARI	merge_except _10Xv3	10Xv3	95.62	0.44	96.17	94.95	95.52	95.74	95.70
scGraphformer	ARI	merge_except _10Xv2	10Xv2	92.07	0.62	92.32	91.69	92.62	92.55	91.17
scGraphformer	ARI	merge_except _Seq-Well	Seq-Well	95.56	0.22	95.84	95.40	95.64	95.66	95.28
scGraphformer	ARI	merge_except _Smart-Seq2	Smart-Seq2	96.91	0.32	97.17	96.67	97.14	97.09	96.47
scGraphformer	ARI	merge_except _CEL-Seq	CEL-Seq	95.84	0.27	95.83	95.55	95.99	95.60	96.21
scGraphformer	ARI	merge_except _Drop-Seq	Drop-Seq	91.97	0.71	92.66	91.51	92.82	91.41	91.45
scGraphformer	ARI	merge_except _inDrop	inDrop	93.95	0.23	93.98	94.23	94.08	93.83	93.63

Table E13 Evaluation comparison while training on Zeisel and annotate Rosenberg.

reference_query	method	mean accuracy	std accuracy
zeisel_rosenberg	scGraphformer	95.21	0.71
zeisel_rosenberg	scmap-cell	94.40	0
zeisel_rosenberg	scVI	91.815	1.144
zeisel_rosenberg	CellTypist	86.642	0.143
zeisel_rosenberg	scBalance	91.633	0.603
zeisel_rosenberg	scmap-cluster	92.53	0
zeisel_rosenberg	ACTINN	92.26	0.45

Table F14 Comparison of methods on Zheng 68K using F1-score.

model	mean value	std value	value[0]	value[1]	value[2]	value[3]	value[4]
scGraphformer	74.95	0.45	75.71	74.35	75.07	74.81	74.79
scVI	69.86	1.07	69.17	68.61	70.41	71.65	69.47
CellTypist	72.15	0.16	72.34	72.17	72.08	72.28	71.89
scBalance	61.96	2.57	58.46	66.03	60.11	62.58	62.61
scmap-cluster	62.58	0.22	62.49	62.89	62.31	62.78	62.42
scmap-cell	54.26	0.63	54.53	53.75	53.68	55.37	53.97
ACTINN	19.20	5.70	14.20	24.72	14.50	15.14	27.46
scBert	74.94	0.57	74.31	75.29	74.31	75.03	75.77
scType	32.36	0.00	32.36	32.36	32.36	32.36	32.36
TOSICA	56.22	0.00	56.22	56.22	56.22	56.22	56.22

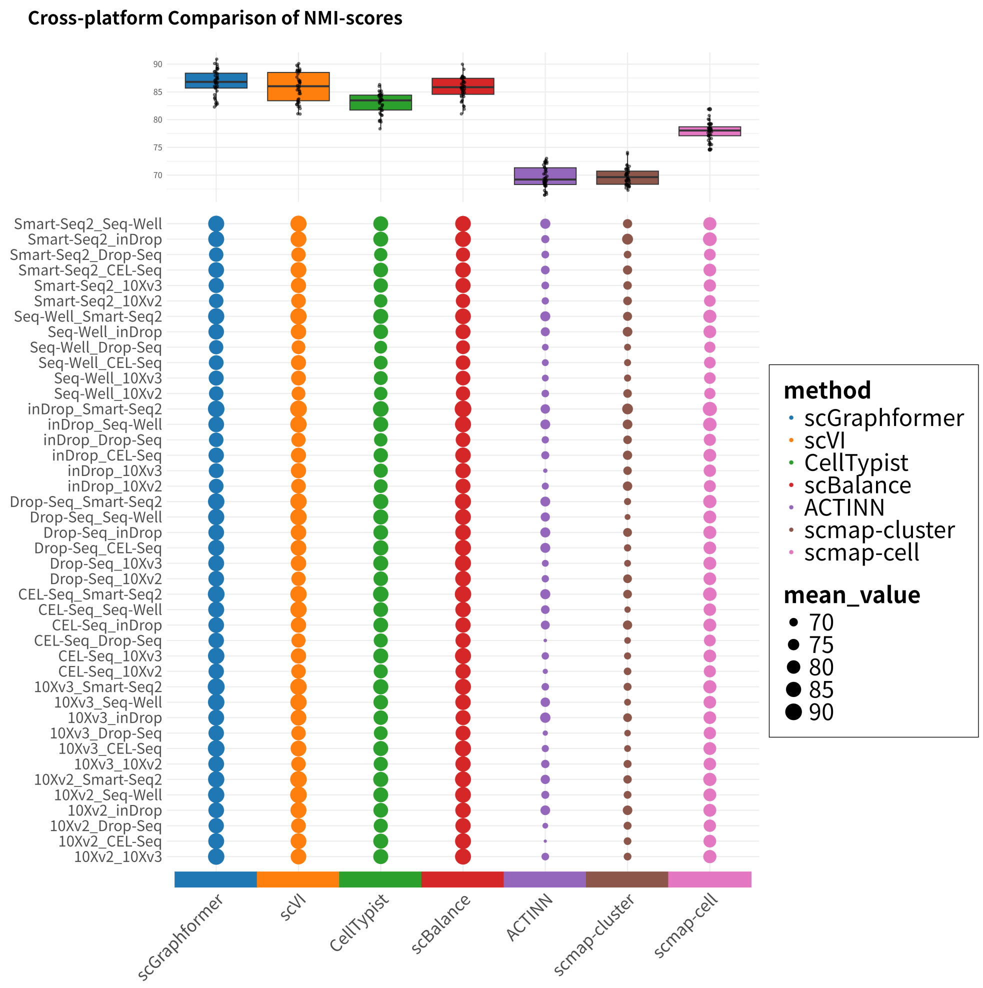

Fig. D3 Comparison on cross-platform experiments using NMI Score metric.

has learned the best cell graph representation. And we guess if there if any biological graph, the situation may be different. But in our article, we still choose to use the results by adding kNN graph since we believe our model could achieve more when we add the real biological graph.

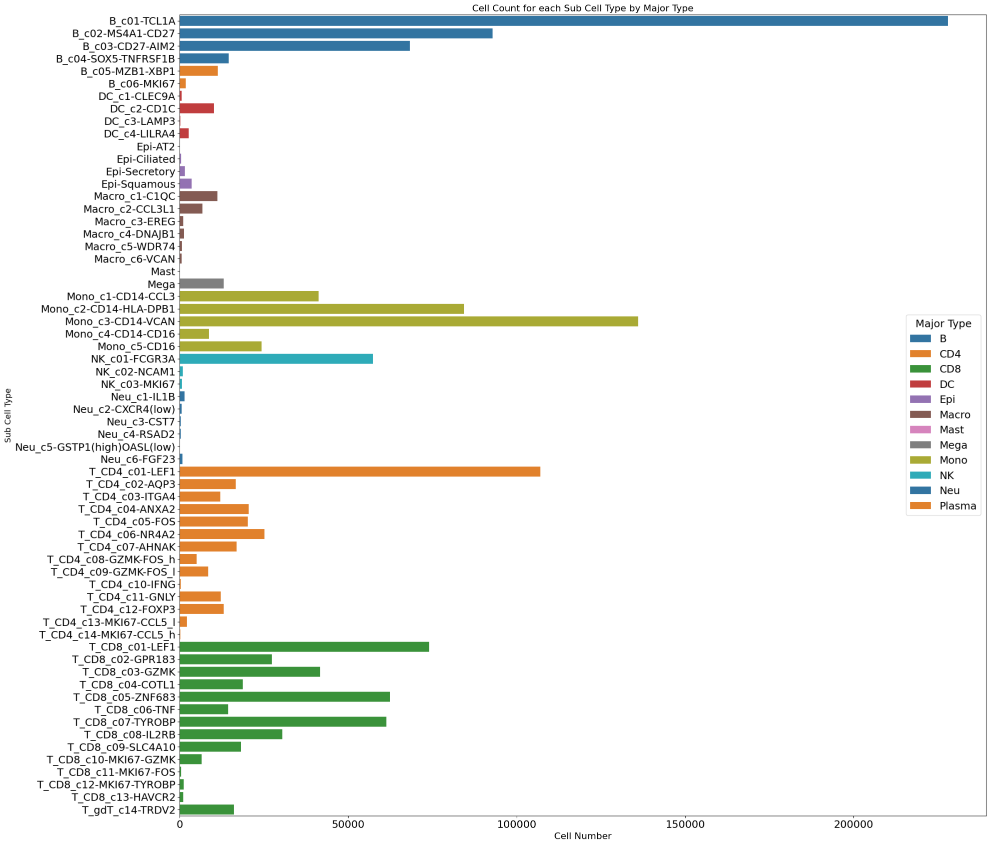

Fig. F4 This figure illustrates the cell type distribution within the COVID-19 Atlas, showcasing the diversity and prevalence of each subtype. Here, each bar represents a specific sub-cell type, with a total of 64 unique subtypes used as training labels during our model development. The colors assigned to each bar indicate the major cell type to which each sub-cell type belongs, facilitating an understanding of their broader biological groupings. Each cell concludes its sub-cell types which are more similarity than others. While training, we used sub-cell types as training labels and there are 64 cell types in total.

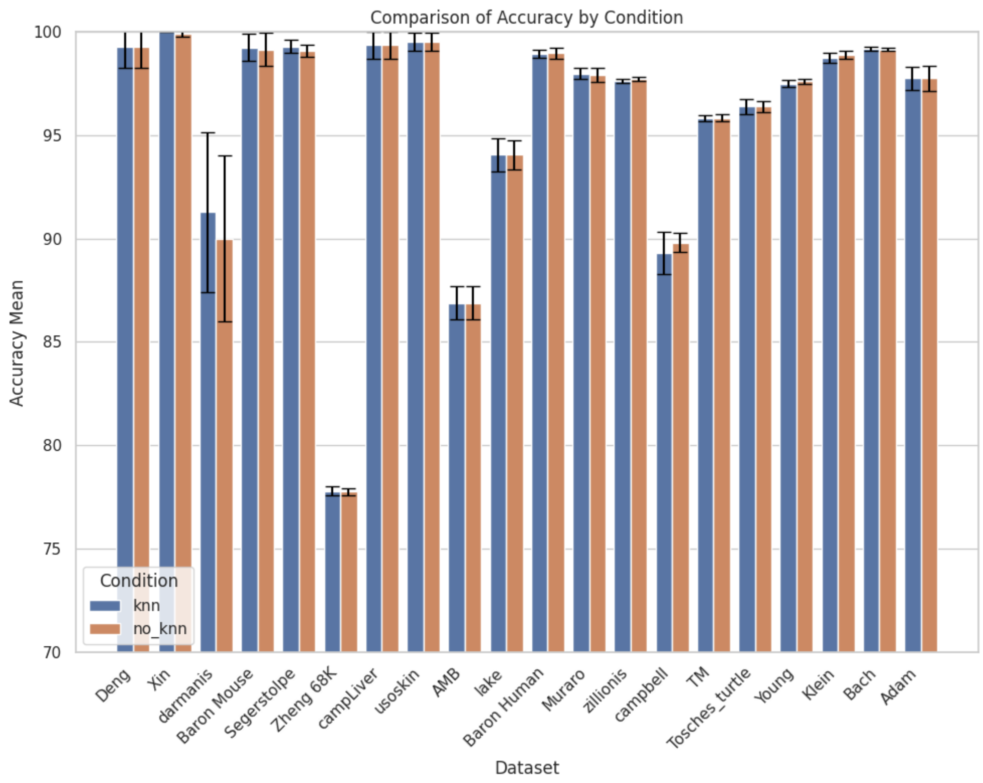

Fig. G5 The performance comparison while training with kNN graph and without no-kNN. In our performance comparison, we investigated the impact of training models with and without a kNN graph. Each experiment was conducted five times to calculate the mean accuracy and standard deviation. The results indicated that incorporating the kNN graph did not significantly alter the outcomes compared to those obtained without using the kNN graph. And we believe this may be because of the non-biological information within the k-NN graph (this may introduce noise in the graph). The all-pair cell network while not adding kNN graph is as efficient as the sparse graph based on kNN graph.

References

[1] Abdelaal, T. *et al.* A comparison of automatic cell identification methods for single-cell RNA-sequencing data (2019). URL https://doi.org/10.5281/zenodo.3357167.

[2] Adam, M., Potter, A. S. & Potter, S. S. Psychrophilic proteases dramatically reduce single-cell rna-seq artifacts: a molecular atlas of kidney development. *Development* **144**, 3625–3632 (2017).

[3] Bach, K. *et al.* Differentiation dynamics of mammary epithelial cells revealed by single-cell rna sequencing. *Nature communications* **8**, 1–11 (2017).

[4] Klein, A. M. *et al.* Droplet barcoding for single-cell transcriptomics applied to embryonic stem cells. *Cell* **161**, 1187–1201 (2015).

[5] Lake, B. B. *et al.* Neuronal subtypes and diversity revealed by single-nucleus rna sequencing of the human brain. *Science* **352**, 1586–1590 (2016).

[6] Tosches, M. A. *et al.* Evolution of pallium, hippocampus, and cortical cell types revealed by single-cell transcriptomics in reptiles. *Science* **360**, 881–888 (2018).

[7] Segerstolpe, Å. *et al.* Single-cell transcriptome profiling of human pancreatic islets in health and type 2 diabetes. *Cell metabolism* **24**, 593–607 (2016).

[8] Young, M. D. *et al.* Single-cell transcriptomes from human kidneys reveal the cellular identity of renal tumors. *science* **361**, 594–599 (2018).

[9] Zheng, G. X. *et al.* Massively parallel digital transcriptional profiling of single cells. *Nature communications* **8**, 14049 (2017).

[10] Deng, Q., Ramsköld, D., Reinius, B. & Sandberg, R. Single-cell rna-seq reveals dynamic, random monoallelic gene expression in mammalian cells. *Science* **343**, 193–196 (2014).

[11] Usoskin, D. *et al.* Unbiased classification of sensory neuron types by large-scale single-cell rna sequencing. *Nature neuroscience* **18**, 145–153 (2015).

[12] Camp, J. G. *et al.* Multilineage communication regulates human liver bud development from pluripotency. *Nature* **546**, 533–538 (2017).

[13] Baron, M. *et al.* A single-cell transcriptomic map of the human and mouse pancreas reveals inter-and intra-cell population structure. *Cell systems* **3**, 346–360 (2016).

[14] Muraro, M. J. *et al.* A single-cell transcriptome atlas of the human pancreas. *Cell systems* **3**, 385–394 (2016).

[15] Tasic, B. *et al.* Shared and distinct transcriptomic cell types across neocortical areas. *Nature* **563**, 72–78 (2018).

[16] Xin, Y. *et al.* Rna sequencing of single human islet cells reveals type 2 diabetes genes. *Cell metabolism* **24**, 608–615 (2016).

[17] Schaum, N. *et al.* Single-cell transcriptomics of 20 mouse organs creates a tabula muris: The tabula muris consortium. *Nature* **562**, 367 (2018).

[18] Campbell, J. N. *et al.* A molecular census of arcuate hypothalamus and median eminence cell types. *Nature neuroscience* **20**, 484–496 (2017).

[19] Zilionis, R. *et al.* Single-cell transcriptomics of human and mouse lung cancers reveals conserved myeloid populations across individuals and species. *Immunity* **50**, 1317–1334 (2019).

[20] Ding, J. *et al.* Systematic comparison of single-cell and single-nucleus rna-sequencing methods. *Nature biotechnology* **38**, 737–746 (2020).

[21] Jorstad, N. L. *et al.* Transcriptomic cytoarchitecture reveals principles of human neocortex organization. *Science* **382**, eadf6812 (2023).

[22] Ren, X. *et al.* Covid-19 immune features revealed by a large-scale single-cell transcriptome atlas. *Cell* **184**, 1895–1913 (2021).

[23] Zeisel, A. *et al.* Molecular architecture of the mouse nervous system. *Cell* **174**, 999–1014 (2018).

[24] Rosenberg, A. B. *et al.* Single-cell profiling of the developing mouse brain and spinal cord with split-pool barcoding. *Science* **360**, 176–182 (2018).
